# A thermosensitive hydrogel encapsulating 2-DG alleviates periodontitis by inhibiting glycolysis and effector response of Th17 cells

**DOI:** 10.3389/fphar.2026.1767931

**Published:** 2026-02-25

**Authors:** Ruowen Zhao, Jia Li, Junhao Yin, Jiabao Xu, Changyu Chen, Jiayu Yan, Siyi Chen, Jiayao Fu, Junhua Wu

**Affiliations:** 1 Department of Prosthodontics, Shanghai Engineering Research Center of Tooth Restoration and Regeneration, Tongji Research Institute of Stomatology, Shanghai Tongji Stomatological Hospital and Dental School, Tongji University, Shanghai, China; 2 Department of Implantology, Shanghai Engineering Research Center of Tooth Restoration and Regeneration, Tongji Research Institute of Stomatology, Shanghai Tongji Stomatological Hospital and Dental School, Tongji University, Shanghai, China; 3 Würzburg Institute of Systems Immunology, Max Planck Research Group, Julius-Maximilians University of Würzburg, Würzburg, Germany; 4 Shanghai Stomatological Hospital, School of Stomatology, Institutes of Biomedical Sciences, Fudan University, Shanghai, China

**Keywords:** 2-DG, hydrogel, metabolism, periodontitis, Th17 cells

## Abstract

**Objective:**

To investigate the mechanism of Th17 cells in immunomodulation during periodontitis and develop a localized drug delivery system based on glycolysis inhibition for safer and more effective therapeutic interventions.

**Methods:**

Periodontitis models were established via the use of IL17A-KO mice to evaluate the impact of Th17-related cytokine deficiency on pathological progression. Using single-cell RNA sequencing (scRNA-seq), we investigated the metabolic profile of CD4^+^ T cells under periodontitis conditions. The glycolysis inhibitor 2-deoxy-D-glucose (2-DG) was used to assess its ability to suppress CD4^+^ T-cell proliferation and Th17 differentiation. A thermosensitive PLGA-PEG-PLGA hydrogel encapsulating 2-DG was synthesized and locally administered to a murine periodontitis model.

**Results:**

IL17A-KO mice exhibited significantly attenuated alveolar bone resorption. Single-cell RNA sequencing revealed that, under periodontitis conditions, CD4^+^ T cells exhibited enhanced differentiation toward Th17 cells and increased glycolysis. The 2-DG hydrogel inhibited CD4^+^ T-cell expansion and Th17 polarization. Local application of the 2-DG hydrogel reduced periodontal inflammation, decreased bone destruction, and diminished granulocyte infiltration in gingival tissues.

**Conclusion:**

Th17-cell differentiation exacerbates periodontitis progression, and glycolysis inhibition effectively modulates Th17-driven immunity. The localized 2-DG hydrogel delivery system presents a promising translational strategy for periodontitis management.

## 1 Introduction

Periodontitis is a prevalent chronic inflammatory disease that affects approximately 11% of the global population (nearly 750 million individuals), with irreversible alveolar bone resorption being the leading cause of tooth loss in adults (Kwon et al., 2021). It is characterized by the destruction of tooth-supporting tissues (gingiva, periodontal ligament, and alveolar bone), manifesting clinically as gingival erythema, tooth mobility, and eventual exfoliation (Hajishengallis, 2015). Recent advances in immunology have highlighted the critical role of immune dysregulation in periodontitis pathogenesis. T cells, particularly CD4^+^ T helper cells (constituting ≈50% of gingival lymphocytes) (Dutzan et al., 2016), are essential for periodontal homeostasis and disease progression. Th17 cells—a subset of CD4^+^ T cells differentiated under retinoic acid receptor-related orphan receptor γt (RORγt) induction (Ivanov et al., 2006)—drive inflammation and bone resorption via IL-17 secretion, neutrophil recruitment, and the production of proinflammatory cytokines (IL6, IL17A, and IL23) (Bunte et al., 2019; Park et al., 2005). Elevated IL17A levels in ligature-induced periodontitis models (Dutzan et al., 2018) and their positive correlation with clinical severity indices (Zenobia and Hajishengallis, 2015) implicate Th17 cells as a therapeutic target, motivating our investigation into the mechanistic role of Th17 cells.

While IL17 receptor knockout (IL17RA KO) mice exhibit exacerbated bone loss after periodontal challenge (Yu et al., 2008) and aggravated periapical infections with elevated neutrophils/macrophages (AlShwaimi et al., 2013), these models are limited by ubiquitous tissue expression of IL17 receptors. In contrast, IL17A knockout (IL17A-KO) mice offer a more precise tool for elucidating the functions of IL17A—an approach rarely employed and thus adopted here.

Cellular metabolism critically regulates T-cell activation and differentiation. The functional specialization of Th subsets, including Th17 cells, is linked to metabolic reprogramming involving glycolysis, fatty acid oxidation, and lipogenesis (Lochner et al., 2015; Kanno et al., 2025; Bantug et al., 2018). Glycolysis is significantly enhanced during naïve CD4^+^ T-cell differentiation toward the Th17 lineage, with Th17 cells relying predominantly on glycolytic flux for energy (Wu et al., 2020; Zhao et al., 2023; Shi et al., 2011). 2-Deoxy-D-glucose (2-DG), a competitive hexokinase inhibitor that blocks the initiation of glycolysis, suppresses macrophage pyroptosis and attenuates periodontitis when it is administered intraperitoneally in LPS-induced models (He et al., 2023). NLRP3, caspase-1, and IL-1β expression in LPS-stimulated macrophages is similarly reduced by HK1 knockdown or 2-DG treatment *in vitro* (Qian et al., 2023). Thus, in addition to genetic ablation of Th17-related cytokines, we pharmacologically inhibited Th17 differentiation via the suppression of glycolysis.

Although systemic 2-DG injection shows preliminary efficacy (He et al., 2023), localized delivery is preferable for periodontitis—site-specific inflammation—to minimize systemic toxicity while maximizing drug concentration. Hydrogels, particularly thermosensitive PLGA-PEG-PLGA copolymers, excel as biocompatible, injectable carriers for periodontal applications (Zheng et al., 2023; Gao et al., 2020; Huang et al., 2023). Capitalizing on these properties, we engineered a 2-DG-loaded hydrogel for local administration to inhibit Th17 polarization and mitigate periodontal destruction.

Overall, this study employs a dual strategy—genetic ablation (IL17A-KO) and pharmacological glycolysis inhibition (2-DG-loaded hydrogel)—to elucidate the role of Th17 cells in periodontitis and establish a targeted local therapy.

## 2 Materials and methods

### 2.1 Bioinformatic analysis of the GEO dataset

This study obtained RNA-seq transcriptome data of periodontal tissue samples (WTBL, n = 4) and healthy periodontal tissue samples (WTBC, n = 4) from the GEO database (GSE244931) via the GEOquery package. The limma package was used to screen for differentially expressed genes (DEGs). With WTBC as the control group, the differential thresholds were set at |log_2_FC| ≥ 1 and an adjusted p value (adj.p.val) <0.05. The results were visualized as a volcano plot via EnhancedVolcano, where red highlights significantly upregulated genes (log_2_FC > 1) in periodontal tissues, blue marks downregulated genes (log_2_FC < −1), and gray indicates genes without statistically significant differences.

Single-cell RNA sequencing data from healthy gingival (HC, n = 4) and periodontitis patient gingival (PD, n = 5) tissues were obtained from the GEO database (GSE171213). Data processing was performed via the Seurat R package (v4.0.5). Quality control was applied with thresholds of 300 < nFeature_RNA < 3,000, nCount_RNA < 15,000, and a mitochondrial gene percentage <25%. The data were then normalized via the “NormalizeData” function, and the top 2000 highly variable genes were identified via “FindVariableFeatures”. Principal component analysis (PCA) was conducted, followed by cell clustering via the “FindClusters” function at a resolution of 0.6, resulting in 16 distinct cell clusters. Cell annotation was performed with the assistance of the CellMarker database. Differential gene expression between the HC and PD groups within CD4^+^ T cells was identified via the “FindMarkers” function. Kyoto Encyclopedia of Genes and Genomes (KEGG) pathway enrichment analysis was performed on the DEGs, and gene set enrichment analysis (GSEA) was specifically applied to the Th17 cell differentiation pathway. These analyses were carried out via the clusterProfiler software package.

### 2.2 Bulk RNA sequencing

CD4^+^ T cells were isolated and purified from mouse spleens via the EasySep™ Mouse CD4^+^ T-Cell Positive Selection Kit II (STEMCELL, Canada). The purified T cells were activated with CD3/CD28 antibodies, with the experimental group supplemented with 20 mM 2-deoxy-D-glucose (2-DG). After 48 h of culture, the cells were collected in TRIzol reagent. RNA sequencing was performed by Personalbio (Shanghai, China) on the Illumina Xplus platform. Differential gene expression analysis was conducted via the DESeq2 R package, and genes with a fold change >1.5 and adjusted p value <0.05 were considered significant. The results were visualized via a heatmap. Kyoto Encyclopedia of Genes and Genomes (KEGG) pathway enrichment analysis was subsequently performed on the DEGs.

### 2.3 Mice and periodontitis (PD) model

C57BL/6 mice were purchased from GemPharmatech (SPF grade). IL17A-IRES-GFP-KI mice (C57BL/6-il17a ^tm1Bcgen^/J, JAX: 018472) and Il17A-KO mice (Cat. No. NM-KO-00131) were obtained from The Jackson Laboratory (USA) and Shanghai Model Organisms Center (China), respectively. All the mice were housed under specific pathogen-free (SPF) conditions at Tongji University Laboratory Animal Center with a 12 h light/12 h dark cycle and provided *ad libitum* access to food and water. Periodontitis models were established in 8-week-old male mice. Ligatures (5–0 silk sutures) were placed bilaterally around the maxillary second molars and retained for 10 days to induce experimental periodontitis. For drug administration, therapeutic compounds were injected every 3 days via sterile insulin syringes until endpoint sampling. All animal procedures were approved by the Ethics Committee (Document No. TJIA2025118).

### 2.4 Cell culture

CD4^+^ T cells and naïve CD4^+^ T cells were isolated from mouse spleens. The culture plates were precoated with an anti-CD3ε antibody (BioLegend, US) overnight at 4 °C. Spleens were mechanically dissociated in PBS containing 2% FBS and filtered through 70-μm nylon mesh to remove clumps and debris. CD4^+^ T cells were purified via the EasySep™ Mouse CD4^+^ Positive Selection Kit II (STEMCELL, Canada), whereas naïve CD4^+^ T cells were isolated via the EasySep™ Mouse Naïve CD4^+^ T-Cell Isolation Kit (STEMCELL, Canada). Purified cells were resuspended in RPMI 1640 medium supplemented with 10% FBS and 1% penicillin‒streptomycin, seeded onto precoated plates, and stimulated with soluble anti-CD28 antibody (BioLegend, US). The cells were cultured at 37 °C under 5% CO_2_ for 48–72 h. For Th17 cell differentiation, naïve CD4^+^ T cells were further treated with TGF-β, IL-6, IL-23, anti-mouse IFN-γ, and anti-mouse IL4. For Th1 cell differentiation, naïve CD4^+^ T cells were further treated with IL-2, IL-12 and anti-mouse IL4. For Th2 cell differentiation, naïve CD4^+^ T cells were further treated with IL-2, IL-4 and anti-mouse IFN-γ.

### 2.5 RNA extraction, reverse transcription, and RT‒qPCR

Total RNA was isolated from samples via TRIzol reagent according to the standard protocol, which involved phase separation with chloroform, RNA precipitation with isopropanol, and washing with 75% ethanol. Reverse transcription was performed with 1,000 ng of RNA using TAKARA’s PrimeScript™ RT Master Mix in a 10 μL reaction. Real-time PCR amplification was performed with SYBR Green Master Mix in 25 μL reactions under the following cycling conditions: initial denaturation at 95 °C for 5 min; 40 cycles of 95 °C for 10 s and 60 °C for 30 s; and a final melting curve analysis to confirm amplification specificity.

### 2.6 Flow cytometry

Fresh cell and tissue samples were processed into single-cell suspensions, and the cells were incubated for 30 min at 4 °C in darkness with the following fluorophore-conjugated antibodies: APC/Cy7-conjugated anti-mouse CD45, PerCP-conjugated anti-mouse/human CD11b, FITC-conjugated anti-mouse F4/80, PE-conjugated anti-mouse F4/80, APC-conjugated anti-mouse Ly6G, FITC-conjugated anti-mouse Ly6G, FITC-conjugated anti-mouse IL17A and APC-conjugated anti-mouse IFN-γ (all from Biolegend). For IL-17A and IFN-γ intracellular staining, the cells were stimulated with PMA and ionomycin for 30 min, treated with brefeldin A for 4 h, fixed and permeabilized. The surface markers were directly stained without prior stimulation. Then, the cells were washed twice with FACS buffer, resuspended in 400 μL of FACS buffer, and subjected to flow cytometry analysis within 4 h. An Annexin V-FITC Apoptosis Detection Kit (Dojindo, Japan) was used for apoptosis detection according to the manufacturer’s instructions. FITC^+^PI^+^ and FITC^−^PI^+^ cells were defined as apoptotic cells. Primary T cells were stained with a CFSE Kit (Thermo, US) following the manufacturer’s protocol. The cells were cultured in an incubator for 72 h, followed by flow cytometry analysis. All flow cytometric analyses were performed by initially gating the major cell population on the FSC-A vs. SSC-A scatter plot to exclude cellular debris; single cells were then selected using the FSC-A vs. FSC-H scatter plot; finally, target cell populations were further gated based on specific markers.

### 2.7 Micro-CT analysis

Mouse maxillae were harvested and scanned via high-resolution micro-CT (μCT50, SCANCO) at a 10 μm spatial resolution at 70 kV/200 μA. SCANCO evaluation software was used to quantify several parameters, including the cementoenamel junction-to-alveolar bone crest distance (CEJ-ABC), bone volume fraction (BV/TV), bone mineral density (BMD) and the bone surface-to-volume ratio (BS/BV). Mimics Medical 21.0 was used to reconstruct three-dimensional images.

### 2.8 Hematoxylin and eosin (H&E) staining

The tissues were fixed in 4% paraformaldehyde for 24 h, paraffin-embedded, and sectioned at 5 μm. After deparaffinization in xylene and rehydration through graded ethanol, the sections were subjected to hematoxylin staining (5 min), differentiation in 1% acid ethanol (10 s), bluing in 0.5% ammonia water (1 min), and eosin counterstaining (10 s). Dehydration via an ethanol series and xylene clearing preceded mounting with neutral resinous medium.

### 2.9 Materials and synthesis of the hydrogel

The thermosensitive PLGA-PEG-PLGA hydrogel (15 wt%) was purchased from Xi’an Ruixi Biological Technology. 2-Deoxy-D-glucose (2-DG) was dissolved in a PLGA-PEG-PLGA triblock copolymer solution at 4 °C to achieve final concentrations of Hy-2-DG of 10 mM, 20 mM, 100 mM, and 500 mM.

### 2.10 Scanning electron microscopy (SEM)

Incubate the hydrogel at 37 °C for 10 min, then quickly transfer it into liquid nitrogen for 5 min. After freezing, move the sample to a lyophilizer and dry for approximately 36 h. Use a blade to fracture the dried hydrogel, perform sputter-coating with gold, and finally observe it under a scanning electron microscope.

### 2.11 Preparation of hydrogel extracts and cell viability assay

The cells were seeded in 96-well plates at a density of 5 × 10^3^ cells/well (n = 5) and cultured for 24 h. Concurrently, the preformed hydrogels and Hy-2-DG were immersed in complete medium at 0.1 g/mL for 24 h at 37 °C to prepare extracts. The following extract formulations were prepared: Hy-25%, Hy-50%, Hy-100%, Hy-2-DG (10 mM)-100%, Hy-2-DG (20 mM)-100% and Hy-2-DG (100 mM)-100%. Following cell attachment, the original culture medium was aspirated and replaced with the respective hydrogel extracts for 24 h of incubation. The extracts were subsequently removed and replaced with freshly prepared CCK-8 solution (Dojindo, Japan), followed by a 2 h incubation at 37 °C. The absorbance at 450 nm was measured via a microplate reader.

### 2.12 Statistical analysis

All the experimental data were statistically analyzed via GraphPad Prism 10. For comparisons between two independent experimental groups, the F test was first performed to assess the homogeneity of variance. If the variances were homogeneous, the unpaired two-tailed Student’s t-test was further conducted. If the variances were heterogeneous, Welch’s t-test was used instead. The data are presented as the means ± standard deviations (SDs). P < 0.05 was considered statistically significant.

## 3 Results

### 3.1 Increased Th17 cell differentiation in the pathogenesis of periodontitis

**FIGURE 1:**
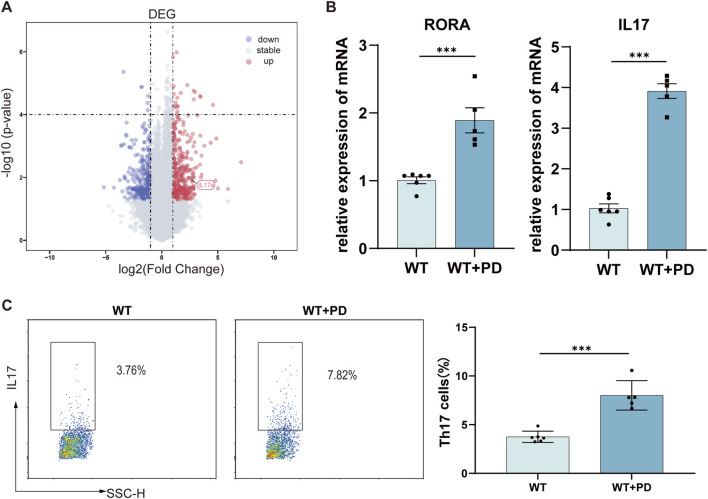
Elevated Th17 cell differentiation in the pathogenesis of periodontitis. **(A)** Volcano plot showing differentially expressed genes (DEGs) in periodontal tissues of ligature-induced periodontitis versus healthy mice. Significantly upregulated (red; log_2_FC > 1, p < 0.05) and downregulated genes (blue; log_2_FC < –1, p < 0.05) are highlighted (GSE244931 dataset). The gray dots represent nonsignificant genes. **(B)** RT‒qPCR analysis of Rora and Il17a mRNA levels in gingival tissues from healthy and periodontitis mice. **(C)** Flow cytometry quantification of Th17 cells in the gingival tissues of periodontitis and control IL17A-IRES-GFP-KI mice. *p < 0.05, **p < 0.01, ***p < 0.001.

Bioinformatic reanalysis of the GSE244931 dataset [11] revealed significantly increased IL17A expression in periodontitis-affected mouse tissues, as demonstrated by a volcano plot (Figure 1A). Consistent with these findings, our murine periodontitis model showed upregulated expression of RORA (a key transcription factor driving Th17 differentiation) and elevated IL17A levels in local gingival tissues (Figure 1B). To precisely track Th17 cells during disease progression, we used IL17A-IRES-GFP-KI mice, in which EGFP expression faithfully reflects IL17A-producing cells. Flow cytometry analysis revealed a marked increase in Th17 cell populations within the gingival tissues of periodontitis mice compared with those of healthy controls (Figure 1C). Collectively, these findings establish a critical role for Th17 cells in periodontitis pathogenesis.

### 3.2 Deletion of Th17-related cytokines ameliorates periodontitis in mice

**FIGURE 2:**
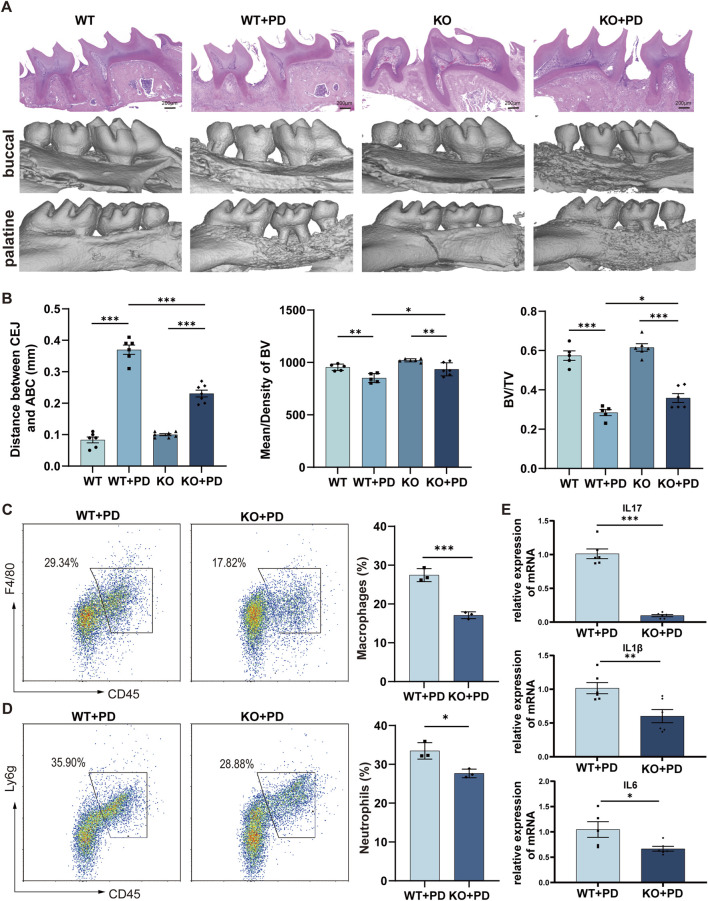
Deletion of Th17-related cytokines ameliorates periodontitis in mice. **(A)** Representative H&E-stained sections and micro-CT 3D reconstructions of periodontal tissues from four experimental groups: healthy wild-type (WT), periodontitis-induced wild-type (WT + PD), IL17A-KO (KO), and periodontitis-induced IL17A-KO (KO + PD) mice. **(B)** Quantitative micro-CT analysis of periodontitis parameters: cementoenamel junction-to-alveolar bone crest (CEJ-ABC) distance, bone volume fraction (BV/TV), and bone mineral density (BMD). **(C,D)** Flow cytometry plots with statistical quantification of macrophage (F4/80^+^CD11b^+^) **(C)** and neutrophil (Ly6G^+^CD11b^+^) **(D)** infiltration in gingival tissues. **(E)** RT‒qPCR quantification of the mRNA expression of inflammatory cytokines (Il17a, Il1b, and Il6) in gingival tissues across the experimental groups. *p < 0.05, **p < 0.01, ***p < 0.001.

In periodontitis, Th17 cells are the major source of IL17A (Dutzan et al., 2016). Periodontitis was induced in both IL17A-KO (KO) and wild-type (WT) mice. Micro-CT and H&E staining analyses consistently demonstrated reduced bone resorption in the KO mice compared with the WT controls. Specifically, deletion of Th17-related cytokines resulted in a shorter distance from the cementoenamel junction to the alveolar bone crest (CEJ-ABC), increased bone volume fraction (BV/TV), and greater bone mineral density (Figures 2A,B). Flow cytometry revealed decreased proportions of neutrophils and macrophages in the gingival tissues of the KO mice (Figures 2C,D), indicating impaired recruitment of these innate immune cells following IL-17A deficiency. Additionally, RT‒qPCR analysis of local gingival tissues revealed decreased expression of key inflammatory cytokines, including IL-1β, IL-17A, and IL-6, in KO mice (Figure 2E). Collectively, these data demonstrate that genetic targeting of Th17 cells effectively alleviates periodontitis.

### 3.3 Glycolysis in CD4^+^ T cells is upregulated in periodontitis

**FIGURE 3:**
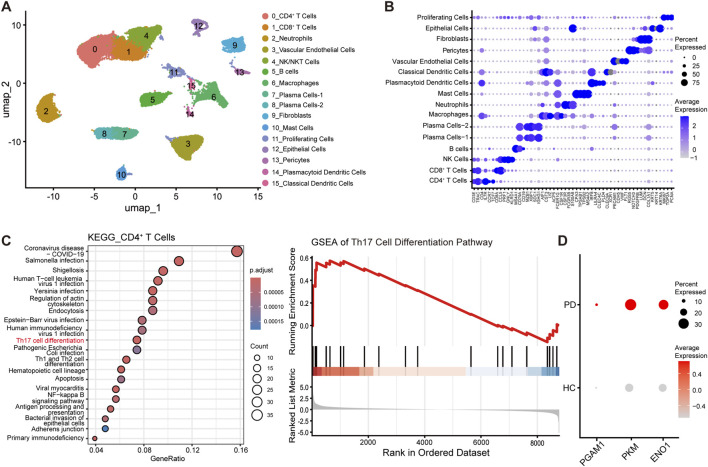
Glycolysis in CD4^+^ T cells is increased in periodontitis. **(A)** UMAP visualization of cells from the GEO dataset (GSE171213) clustered into 16 distinct groups at a resolution of 0.6. **(B)** Dot plot displaying representative markers for the 16 cell clusters. **(C)** KEGG pathway enrichment analysis of CD4^+^ T cells (left) and GSEA of the Th17 cell differentiation pathway (right). **(D)** Dot plot showing the expression levels of glycolysis-related genes in the periodontitis (PD) and healthy control (HC) groups (p < 0.05).

Compared with genetic manipulation, pharmacological inhibition represents a safer therapeutic strategy. Cellular metabolism is intrinsically linked to cell differentiation processes. Analysis of the GEO dataset (GSE171213), which included 4 normal gingival samples and 5 periodontitis gingival samples, clustered all the cells into 16 distinct clusters at a resolution of 0.6 (Figures 3A,B). Further analysis of CD4^+^ T cells revealed significant enrichment of the Th17 cell differentiation pathway among the DEGs in the periodontitis (PD) group compared with the healthy control (HC) group. Gene set enrichment analysis (GSEA) further confirmed positive enrichment of this pathway in the PD group, indicating its significant activation (Figure 3C). Additionally, the expression of glycolysis-related genes (PGAM1, PKM, and ENO1) in CD4^+^ T cells was significantly increased in the PD group (Figure 3D), suggesting enhanced glycolytic activity in periodontitis. This metabolic shift may positively contribute to the observed promotion of Th17 cell differentiation. Beyond this, we examined the expression of glycolysis-related genes in other immune cells. We found that macrophages and CD8^+^ T cells in the periodontitis (PD) group highly expressed genes that promote glycolysis, whereas neutrophils and B cells showed fewer differentially expressed glycolysis-related genes (Supplementary Figure A). This potentially indicates that subsequent therapeutic strategies targeting glycolysis may exert their effects by acting on multiple types of immune cells.

### 3.4 Glycolysis inhibitor-loaded hydrogel suppresses CD4^+^ T-cell proliferation and Th17 differentiation

**FIGURE 4:**
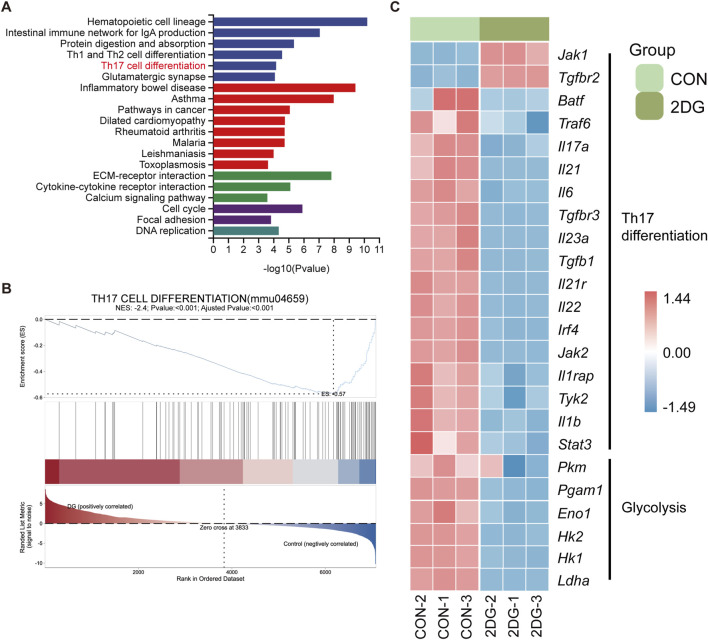
2DG treatment inhibits the differentiation of CD4^+^ T cells toward Th17 cells. **(A)** KEGG pathway enrichment analysis of DEGs between the CON and 2DG-treated groups. **(B)** GSEA plot of the Th17 cell differentiation pathway. **(C)** Heatmap displaying the expression of Th17 differentiation-related genes and glycolysis-related genes in the CON and 2DG-treated groups.

2-DG (2-deoxy-D-glucose) is a glycolysis inhibitor that competitively inhibits glucose metabolism by targeting hexokinase. Given the enhanced glycolysis in CD4^+^ T cells under periodontitis conditions, we treated primary CD4^+^ T cells with 2-DG and performed transcriptome sequencing to further validate the effect of glycolysis inhibition on CD4^+^ T-cell differentiation. KEGG analysis revealed that DEGs in the 2DG-treated group were significantly enriched in the Th17 cell differentiation pathway compared with those in the normal control group (Figure 4A). Furthermore, GSEA revealed negative enrichment of this pathway in the 2DG-treated group (Figure 4B), with most pro-Th17 differentiation genes being significantly downregulated following 2DG treatment (Figure 4C).

**FIGURE 5:**
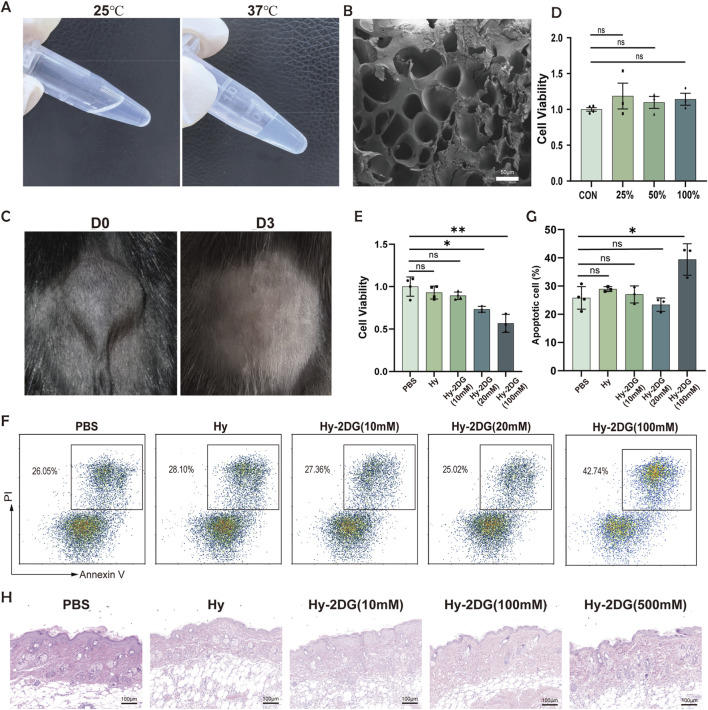
Characterization of PLGA-PEG-PLGA hydrogel. **(A)** Representative images documenting the sol‒gel transition process. **(B)** SEM image of the hydrogel. **(C)** Macroscopic appearance of the subcutaneously implanted hydrogel immediately after injection (D0) and after 72 h of degradation (D3). **(D)** CCK-8 viability assay of cells treated with serial dilutions of blank hydrogel extract (25%, 50%, 100%). **(E)** CCK-8 viability assay of the PBS control, blank hydrogel group (Hy), and 2-DG-loaded hydrogel extracts (Hy-2-DG (10 mM), Hy-2-DG (20 mM), Hy-2-DG (100 mM)). **(F,G)** Flow cytometry plots showing the quantification of apoptosis in CD4^+^ T cells across groups: PBS control, blank hydrogel (Hy), Hy-2-DG (10 mM), Hy-2-DG (20 mM) and Hy-2-DG (100 mM). **(H)** H&E-stained sections of subcutaneous tissues 72 h post-injection with PBS control, blank hydrogel (Hy), and 2-DG-loaded hydrogels at increasing concentrations (Hy-2-DG (10 mM), Hy-2-DG (100 mM), Hy-2-DG (500 mM)). *p < 0.05, **p < 0.01, ***p < 0.001.

To increase intraoral drug retention, we utilized an experimentally validated injectable thermosensitive PLGA-PEG-PLGA hydrogel as the delivery vehicle. This hydrogel exhibited rapid sol‒gel transition within 5 min at 37 °C (Figure 5A). Scanning electron microscopy (SEM) images of the vacuum freeze-dried hydrogel revealed a three-dimensional continuous porous network (Figure 5B). Furthermore, the hydrogel completely degraded within 72 h after subcutaneous injection in mice (Figure 5C). Biosafety assessments revealed the following: 1) The CCK-8 assay revealed that gel extracts at various concentrations did not affect cell viability (Figure 5D), whereas the hydrogel loaded with 2-deoxy-D-glucose (2DG) at a high concentration (100 mM) led to a reduction in cell viability (Figure 5E). 2) Apoptosis assays indicated that the hydrogel loaded with a low concentration of 2DG had no significant effect on apoptosis, while treatment with the Hy-2DG (100 mM) group resulted in increased apoptosis (Figures 5F,G). 3) Considering that factors such as biological barriers and pharmacokinetics can reduce drug availability at the target site (Jiang et al., 2026), we employed hydrogels with higher concentration gradients for subcutaneous injection and performed HE staining to evaluate *in vivo* biocompatibility. The results indicated no significant adverse reactions (e.g., rejection or inflammation) (Figure 5H). Collectively, these results establish the favorable biocompatibility, timely biodegradability, and minimal immunogenicity of this hydrogel system.

**FIGURE 6:**
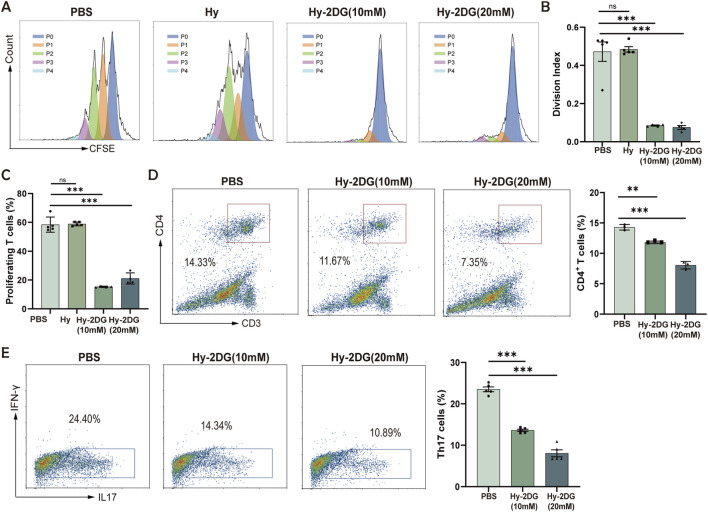
Hy-2-DG suppresses CD4^+^ T-cell proliferation and Th17 polarization. **(A–C)** CFSE analysis of CD4^+^ T cells exposed to indicated treatments, including statistical analysis of division index **(B)** and percentage of proliferating cells **(C,D)** Flow cytometry quantification of CD4^+^ T cells proportions in the PBS, Hy-2-DG (10 mM), and Hy-2-DG (20 mM) groups. **(E)** Flow cytometry quantification of Th17 cell proportions following *in vitro* polarization of naïve CD4^+^ T cells under Th17-inducing conditions in the PBS, Hy-2-DG (10 mM), and Hy-2-DG (20 mM) groups. *p < 0.05, **p < 0.01, ***p < 0.001.

Further investigations into the functional impact of Hy-2-DG on T-cell differentiation revealed critical insights. Flow cytometric analysis confirmed that Hy-2-DG significantly suppressed CD4^+^ T cell proliferation and division, as measured by CFSE assays (Figures 6A–C), concurrently reducing the proportion of CD4^+^ T cells and inhibiting the polarization of naïve CD4^+^ T cells toward the Th17 lineage (Figures 6D,E). These findings at the cellular level demonstrated the potent inhibition of Th17 differentiation by the hydrogel system, suggesting its therapeutic potential for periodontitis management. In addition, we further investigated its effects on the proportions of other immune cells. It was found that Hy-2-DG could also markedly reduce the proportions of CD8^+^ T cells, and slightly decrease the proportion of Th1 cells (Supplementary Figure B–D).

### 3.5 Localized 2-DG-loaded hydrogel application attenuates periodontitis progression

**FIGURE 7:**
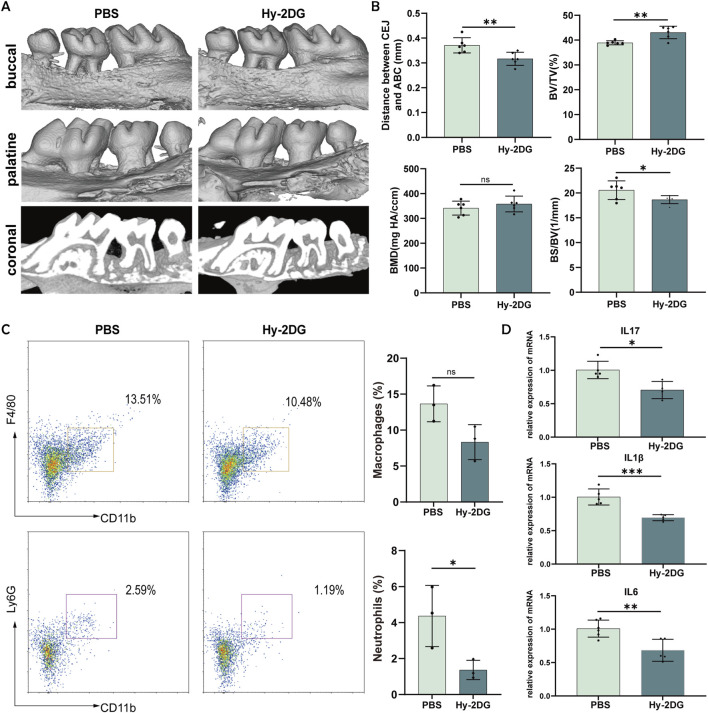
Local application of the 2-DG-loaded hydrogel attenuated periodontitis. **(A)** Micro-CT 3D reconstructions and coronal sections of periodontal tissues from wild-type mice with ligature-induced periodontitis treated with PBS or Hy-2-DG. **(B)** Quantitative analysis of the following micro-CT parameters: cementoenamel junction-to-alveolar bone crest (CEJ-ABC) distance, bone volume fraction (BV/TV), bone surface/volume ratio (BS/BV), and bone mineral density (BMD). **(C)** Flow cytometry plots and quantification of macrophage and neutrophil proportions in gingival tissues. **(D)** Inflammatory cytokine expression (Il1b, Il17a, and Il6) in gingival tissues was measured by RT‒qPCR. *p < 0.05, **p < 0.01, ***p < 0.001.

Following *in vitro* validation of the immunomodulatory effects of Hy-2-DG, we evaluated its therapeutic efficacy in ligature-induced murine periodontitis. The experimental groups received localized gingival injections of Hy-2-DG (100 mM) every 3 days, whereas the control groups received saline. Micro-CT analysis demonstrated significant mitigation of alveolar bone resorption in Hy-2-DG-treated mice, as evidenced by a reduced CEJ-ABC distance, increased bone volume fraction (BV/TV) and decreased bone surface-to-volume ratio (BS/BV) (Figures 7A,B). Flow cytometric quantification revealed decreased neutrophil infiltration and a trending reduction in macrophage populations within the gingival tissues of treated mice (Figure 7C). Similarly, RT‒qPCR analysis confirmed the downregulation of key inflammatory mediators (IL-1β, IL-6, and TNF-α) in periodontal tissues (Figure 7D). These *in vivo* results establish that localized Hy-2-DG administration exerts potent anti-inflammatory effects and attenuates pathological bone resorption in periodontitis.

## 4 Discussion

Our findings elucidate the pivotal role of Th17 cells in periodontitis progression, as evidenced by significantly reduced alveolar bone resorption in IL17A-KO mice. We further employed the glycolysis inhibitor 2-DG to suppress Th17 differentiation in CD4^+^ T cells and pioneered a localized delivery strategy using a thermosensitive PLGA-PEG-PLGA hydrogel for sustained 2-DG release. This system effectively mitigated bone destruction and granulocyte infiltration in periodontitis models, establishing a novel immunometabolic-targeted therapeutic approach.

Reduced granulocyte infiltration upon IL-17A deficiency or 2-DG treatment aligns with established mechanisms: IL17A induces fibroblast-derived CXCL1 to recruit granulocytes (Su et al., 2025) and stimulates epithelial/stromal cells to secrete granulocyte colony-stimulating factor (G-CSF), promoting pathogenic neutrophil differentiation (Hong et al., 2025). While granulocyte reduction is correlated with disease attenuation, the dual roles of immune cells in inflammation warrant consideration. Neutrophils and macrophages clear pathogens and initiate tissue repair but may exacerbate inflammation when hyperactivated (Guan et al., 2025; Fantone et al., 2025). This delicate balance remains incompletely understood. Moreover, inflammation resolution depends not only on cell numbers but also on functional states and polarization (Guan et al., 2025; Ng et al., 2025)—the mechanisms underlying IL-17A deficiency and 2-DG treatment require further investigation.

Notably, although IL17A knockout alleviates periodontitis, IL17RA deficiency exacerbates it (Yu et al., 2008; AlShwaimi et al., 2013). This may partially resolve the aforementioned discrepancy. In addition to IL17A, the IL17 cytokine family comprises IL17B, IL17C, IL17D, IL17E, and IL17F (Kolls and Linden, 2004). Functionally, IL17F promotes proinflammatory responses analogous to those of IL17A (Vidal et al., 2022), while IL17C activates innate immune pathways in epithelial cells through autocrine stimulation, inducing the expression of cytokines, chemokines, and antimicrobial peptides (Swedik et al., 2021). However, it should be emphasized that IL-17A knockout does not eliminate Th17 cells, which can still produce IL-17F and other cytokines. These residual Th17-derived cytokines may exert regulatory effects through shared receptor pathways, potentially influencing the observed phenotypes. This represents a limitation of our study that warrants further investigation. Moreovre, IL17RA is ubiquitously expressed across tissues (Moseley et al., 2003). Although the IL17 receptor family comprises five subunits (IL17RA, IL17RB, IL17RC, IL17RD, and IL17RE), IL17RA serves as an obligatory subunit shared by other IL17 receptors and is indispensable for signal transduction of all IL17 cytokines except IL17D (Vidal et al., 2021). Reported evidence indicates that IL17RA deficiency predisposes hosts to bacterial and fungal infections (Levy et al., 2016), and IL17RA-deficient mice exhibit impaired neutrophil migration to bone tissues (Yu et al., 2007). Consequently, with respect to immune cell recruitment alone, IL17RA ablation may have more profound consequences than does IL17A knockout, resulting in systemic immune dysregulation and exacerbated pathology. In contrast, selective IL17A depletion—while effectively blocking downstream inflammatory cascades—exerts minimal impact on fundamental immune surveillance.

Currently, PLGA-PEG-PLGA thermosensitive injectable hydrogels have been extensively utilized as drug delivery carriers (Gao et al., 2020; Huang et al., 2023; Zhu et al., 2024). These hydrogels exhibit excellent biocompatibility and biodegradability, as they undergo degradation *in vivo* through enzymatic and nonenzymatic hydrolysis into PLGA and PEG, with PLGA further breaking down into water and carbon dioxide (Makadia and Siegel, 2011). Moreover, local injection circumvents systemic administration, thereby significantly reducing the risk of systemic exposure. This study is the first to employ PLGA-PEG-PLGA thermosensitive hydrogels for periodontal-specific 2-DG delivery. Conclusion: Combining localized IL-17A signaling blockade with glycolysis inhibition pioneers safe and effective immunometabolic therapy for periodontitis.

## Data Availability

Publicly available datasets were analyzed in this study. This data can be found here: NCBI (GEO) repository, accession number GSE171213 (https://www.ncbi.nlm.nih.gov/search/all/?term=GSE171213).
