# Supplementary material for: A thermosensitive hydrogel encapsulating 2-DG alleviates periodontitis by inhibiting glycolysis and effector response of Th17 cells
Source: Front Pharmacol. 2026 Feb 25;17:1767931. doi: 10.3389/fphar.2026.1767931 (PMC12975590; doi:10.3389/fphar.2026.1767931)
Supplement: Supplementary file 1 [file DataSheet1.doc]

**Supplementary Figure**


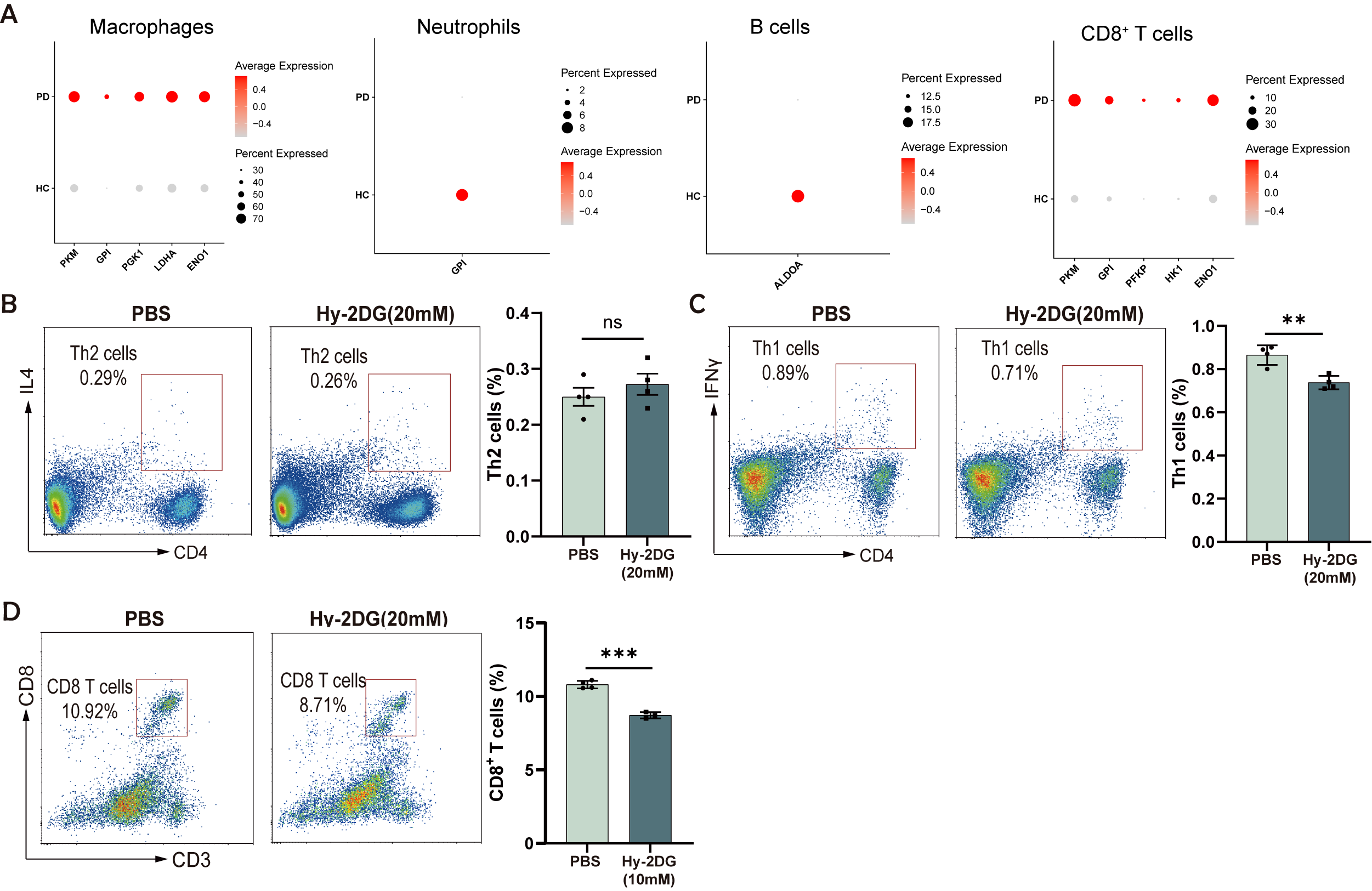


Supplementary Figure. (A) Dot plots depicting the expression of glycolysis-related genes in macrophages, neutrophils, B cells, and CD8⁺ T cells, comparing the periodontitis (PD) and healthy control (HC) groups (p < 0.05). (B-D) Changes in the proportions of Th2 (B), Th1 (C) and CD8⁺ T cells (D) under different treatment conditions.
